# Depression in persons with disabilities: a scoping review

**DOI:** 10.3389/fpubh.2024.1383078

**Published:** 2024-05-07

**Authors:** Syed Mohammed Basheeruddin Asdaq, Sultan Alshehri, Sarah Abdulrahman Alajlan, Abdulaziz Ali Almutiri, Abdullah Khatim R. Alanazi

**Affiliations:** Department of Pharmacy Practice, College of Pharmacy, AlMaarefa University, Ad Diriyah, Saudi Arabia

**Keywords:** cognitive behavior therapy, depression, disability, physical activity, psychological counseling, public health, scoping review

## Abstract

Individuals with disabilities are more vulnerable to depression development than the general population. This study sought to map the evidence on current knowledge of depression, intervention strategies, and assessment tools among people with disabilities. This review was conducted following Arksey and O′Malley’s scoping review methodology framework. An electronic search was performed on four English databases: PubMed, Cochrane Library, PsycINFO, and Web of Science. The original search returned 1802 results, with 1,116 from Web of Science, 626 from PubMed, 25 from Cochrane, and 35 from PsycINFO. After removing duplicates, 786 articles were chosen for the title and abstract screening processes. Finally, 112 full-text publications were deemed eligible, with 41 papers being included in this scoping review for analysis. A large proportion (32; 78.04%) of the studies chosen were cross-sectional, 14 (34.14%) of them reported general disability, 12 (29.26%) used a patient health questionnaire (PHQ-9) to measure depression, and 14 (34.14%) had interventions, including cognitive behavioral therapy, psychological counseling, social support, and physical activity. All interventions successfully reduced the severity of the depression. Cognitive behavioral therapies and psychological counseling were widely used interventions that had a significant impact on reducing depression. More randomized controlled trials are required, and they should focus on individuals with specific disabilities to provide disability-specific care that can improve the quality of life for disabled individuals.

## Introduction

A disability is defined as any physical or mental defect (impairment) that makes it difficult for the affected individual to engage in activities (activity limitation) or interact with others (participation limits) (1). This can result in the absence of significant differences in a person’s body or mental functioning (2). The World Health Organization first released the International Classification of Functioning, Disability, and Health (ICF) in 2001. The ICF provides a common vocabulary for defining bodily functions and structures, levels of activity and participation, and environmental situations that influence an individual’s health.

Disability is a major concern in public health not only because it affects a large proportion of the world’s population, but also because most people will face the challenge of living with a disability at some point in their lives (3). To ensure that people with disabilities have equal access to community life and can participate in daily tasks and responsibilities, communities must adapt their physical and social environments.

The Disability and Health Data System (DHDS), an online data source for adults with disabilities in the United States, monitors, and records six different types of functional disabilities (4). These disorders impair a person’s ability to carry out their daily activities. They include cognitive impairment (serious difficulty focusing, remembering, or making decisions), hearing impairment (serious difficulty hearing or deafness), mobility impairment (serious difficulty walking or climbing stairs), vision impairment (serious difficulty seeing or blindness), self-care impairment (difficulty dressing or bathing), and independent living impairment (difficulty running errands alone).

According to recent estimates, more than 1.3 billion people live with significant disabilities, accounting for 16% of the global population. This figure is rising as noncommunicable diseases become more prevalent and the average human lifespan increases. People with disabilities are a diverse group whose life experiences and healthcare needs are influenced by a variety of factors such as gender, age, gender identity, sexual orientation, religion, race, ethnicity, and socioeconomic status. People with disabilities have shorter lifespans, poorer health, and more difficulty performing daily tasks than the general population (5). According to a study published by the Centers for Disease Control and Prevention (CDC), the percentage of adults with impairments was highest in noncore (rural) counties and lowest in big, central, and fringe metropolitan (urban). Adults in noncore counties were 9% more likely to report a disability, 24% more likely to report three or more disabilities, 7% more likely to report a cognitive disability, and 35% more likely to report a hearing disability (6). Rural communities often have less access to clinics and hospitals, are more geographically isolated, and have fewer transportation options than urban areas (7). As a result, access to public health programs that include people with disabilities is critical and should be equally present in all habitats.

The 2017 Saudi disability survey (8) reports that the proportion of Saudis with disabilities (mild, severe, and extreme) is 7.1% of the total population, with men making up 3.7% and women 3.4%. In contrast, a study based on the 2013 survey in the United States reported a 22.2% disability rate, with women having a higher prevalence rate of 24.4% than men (19.8%) (9). In Saudi Arabia, it is estimated that 3.1% of the population has multiple disabilities, with 1.5% of the male population and 1.6% of the female population. According to the 2013 US survey, Alabama states had a disability rate of 31.5%, while the Riyadh region had the highest percentage of disabled Saudis (25.13% of the total Saudi population with disabilities). Mobility impairment (difficulty walking and climbing stairs) and vision impairment (severe vision problems or blindness) were the two most common significant disabilities found in the Saudi population, representing 4.1 and 4% of the population, respectively. In the United States, the most reported type of disability was mobility (13.0%), which was followed by cognition (10.6%), independent living (6.5%), vision (4.6%), and self-care (3.6%) (9).

The population group of children with disabilities is extremely diverse. These comprise kids who were born with a hereditary disorder that impacts their physical, mental, or social development; those who had a major accident, malnutrition, or infection that had an impact on their long-term functioning; and those who were exposed to pollutants in the environment that caused learning disabilities or developmental delays. Children 18 years of age or younger who have “long-term physical, mental, intellectual, or sensory impairments that, in combination with various barriers, may hinder their full and effective participation in society on an equal basis with others” are considered disabled according to the 2006 Convention on the Rights of Persons with Disabilities (CRPD; 10). Based on home surveys of children’s functional status, United Nations Children’s Fund (UNICEF) estimated that 28.9 million (4.3%) children worldwide have moderate-to-severe disabilities, as do 207.4 million (12.5%) children aged 5–17 years and 236.4 million (10.1%) children aged 0–17 years (10).

People with disabilities are typically excluded from mental health research. This is despite strong evidence linking disability to depression (11) for a variety of reasons, including decreased autonomy, a lack of social support, and biochemical pathways (12–15).

Different types of disabilities, such as learning (16), sensory (17), and neurodegenerative (18), have been linked to a higher prevalence of depression or depressive symptoms than the general population. There is evidence of a complex bidirectional causal pathway, particularly between physical impairment and depressive symptoms: depressive symptoms can result in functional limitations, with those who are depressed reporting greater difficulty performing daily activities. Physical limitations can cause or exacerbate depressive symptoms (11, 19–21). People with physical limitations who experience depressive symptoms also experience pain in a bidirectional form (22). Severe pain can lead to increased levels of depressive symptoms, which can lead to worse pain.

Yang (23) examined longitudinal data from the United States and discovered a significant link between functional disability and elevated depression symptoms in the older adult population. Although social support may help to mitigate some of these effects, disability still has a significant impact on depression. Barry et al. (24) discovered an association between disability severity and depression symptoms in older people.

Most studies (25, 26) have concentrated on defining the clinical presentation of depression in the disabled population. The incidence of depression varies depending on the approach. Depression was reported or diagnosed in 25 to 44% of people with developmental disabilities (26, 27). Research on the prevalence of depression in people with traumatic brain injury has been inconsistent, with estimates ranging from 6 to 77%. This is partly determined by the verification strategy used in the study. These methods included self-reporting, the use of standardized assessment tools, interviews, and clinical record diagnoses (28, 29). Depressive disorder was diagnosed in 27% of the 650 patients evaluated by the Traumatic Brain Injury Model Systems programs (30, 31). A study of rheumatoid arthritis patients discovered that 39.2% of the participants were depressed (32). A systematic review article on the relationship between stroke and depression included 14 studies and found that depression prevalence was 19–21% 2 years after stroke (33). According to Deb et al. (34), only 2.2% of their sample with mild to severe intellectual disabilities was depressed; however, McGillivray and McCabe (35) revised this figure to 39%. Hermans et al. (36) reported a 5% prevalence of depression, while Hermans et al. (37) reported a 7.6% prevalence of depression in older adult and disabled people.

Generally, the prevalence rates of depression for any specific disability condition were found to vary significantly between studies. The wide range of reported prevalence rates can be attributed to methodological differences and complexities. As a result, it is difficult to compare findings from various studies on the subject. A variety of measuring devices have been used to detect depression. Furthermore, variation in the study sample is likely the most significant factor contributing to the large disparity in prevalence estimates. People who live in institutions may be different from those who live with their families, with the latter having the highest prevalence rates (38). Age and degree of impairment are two other factors that can influence the severity of depression. According to Hermans et al. (37), there appears to be a link between aging and increased depression symptoms. Research suggests that individuals with mild to moderate disabilities are more likely than those with severe disabilities to experience depression (39, 40). However, the data cannot be easily compared or synthesized due to the variety of measurement and reporting methodologies used in research.

Taking these factors into consideration, a scoping review is a better option. It can effectively summarize the current state of research on a specific topic, demonstrating its breadth, depth, and limitations while also providing new information for the future. As a result, the purpose of this scoping review is to identify the disabilities that are most frequently associated with depression. We also looked at the assessment method and sociodemographic factors that may influence the expression of depressive symptoms in people with disabilities, as well as some of the therapies or strategies documented in the literature as interventions for managing depression in the disabled population.

## Methods

The study was prepared using Arksey and O'Malley's (41) reference framework. We followed a five-step process: (1) formulate the research question, (2) locate relevant studies, (3) select studies, (4) chart data, and (5) summarize and report the findings. The Preferred Reporting Items for Systematic Reviews and Meta-Analyses (PRISMA) Scoping Review standards were used to report this scoping review (42).

### Formulate the research questions

The major questions for this scoping review were: (a) What types of disabilities are associated with depression? (b) How is depression assessed among people with disabilities? (c) What are the strategies/interventions for dealing with depression in people with disabilities?

### Find relevant studies

We searched four English-language databases for relevant publications published between 2019 and August 2023, including PubMed, Cochrane Library, PsycINFO, and Web of Science. The search strategy used the phrases “disability” and “depression,” as well as all relevant keywords, mesh terms, and index terms. Combinations of these words with relevant synonyms were also considered. Only primary research articles involving either retrospective or prospective data were included in the study, whereas review articles were not included. The search is limited to English-language publications that deal with human studies.

### Study selection

The following were the inclusion criteria: (1) Persons with disabilities (all ages) were the intended audience; (2) Depression was the main dependent or independent variable; and (3) The articles would be published in English. The following were the conditions for exclusion: (1) Online papers that are not published in scientific journals, such as workshop abstracts; (2) editorial papers and opinion articles; (3) Internet brochures or advertisements; (4) non-human research; and (5) review article.

### Data charting

We used Reference Manager to manage, categorize, sort, retrieve, and edit references. Following the removal of duplicate records, two researchers independently evaluated the titles and abstracts of papers using the inclusion and exclusion criteria. Following that, they read the entire text to determine which references should be the final ones added to the study. In the event of a disagreement, a third researcher joined the discussion and provided feedback. The following fields were used to obtain the final data from the included studies: (1) Basic information, including the author, country, and publication date. (2) Study features include design, setting, goals, sample demographics, and results. (3) the type of disability identified; (4) an assessment tool for measuring depression; and (5) interventions and methods for managing depression. The included studies were summarized using descriptive methods, and the results were reported as numbers and percentages. Additionally, the risk of bias assessment is not applicable due to the nature of the scoping review itself.

## Results

### Articles retrieved

**Figure 1:**
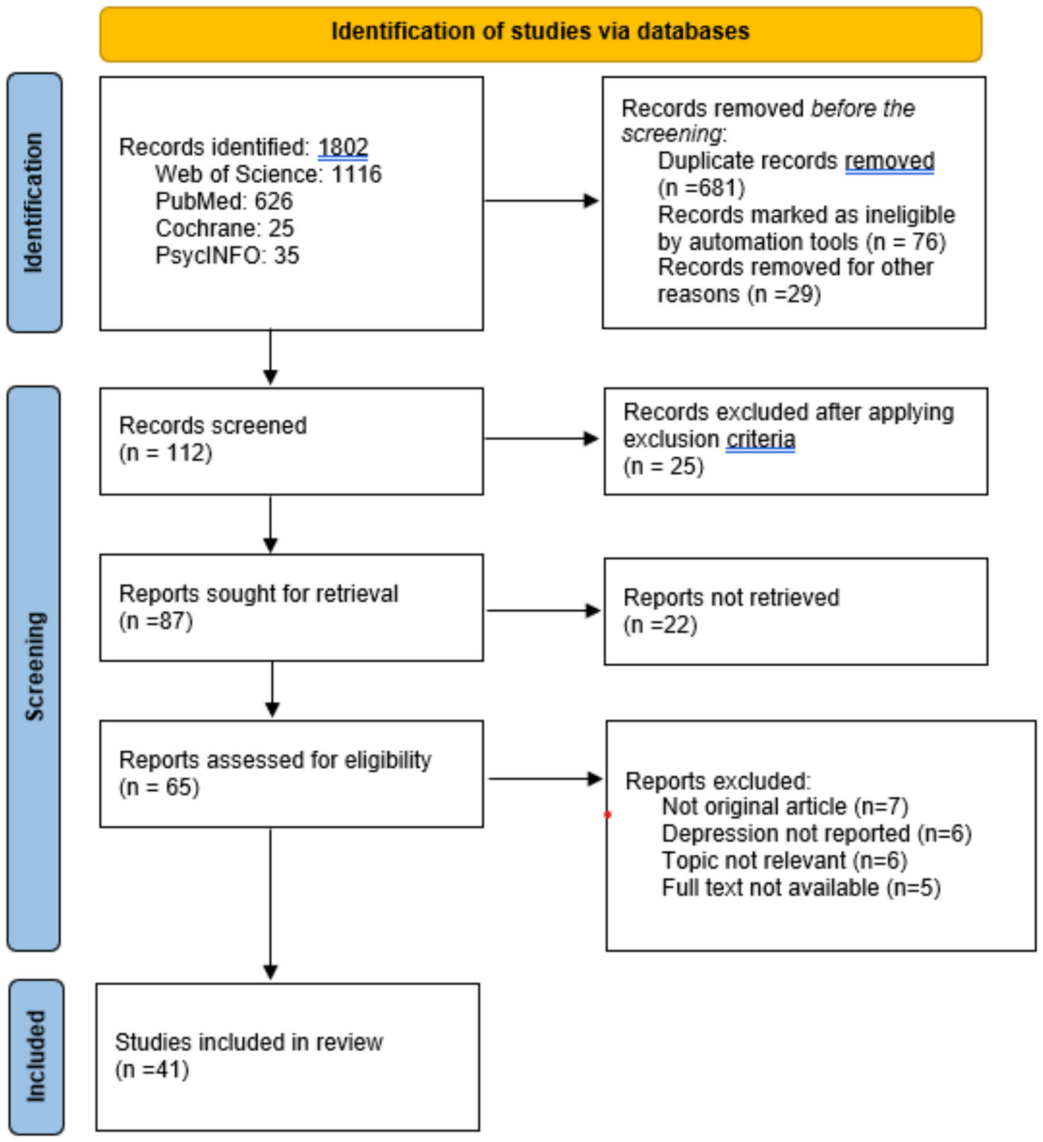
PRISMA diagram for selected studies in the review.

All literature searches had been completed by August 2023. The original search yielded 1802 results, including 1,116 from the Web of Science, 626 from PubMed, 25 from Cochrane, and 35 from PsycINFO. After removing duplicates, 786 articles were selected for the title and abstract screening process. Finally, 112 full-text publications were considered eligible, and 41 papers were included in this scoping review for analysis. Figure 1 depicts the PRISMA diagram-based procedures used in the identification, screening, and inclusion steps.

### Characteristics of selected articles

**Figure 2:**
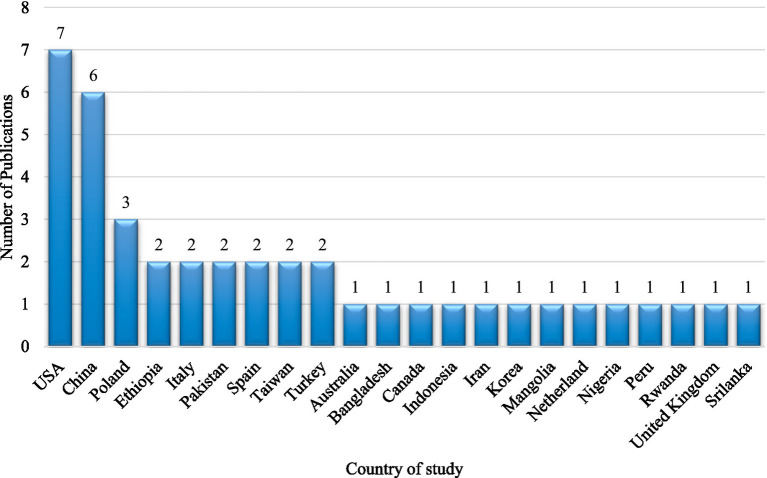
Statistics of country of study.

The publications in this study are based on research conducted in 22 countries. Many studies on the nature of depression in individuals with disabilities have been conducted in the United States of America. The data analysis included seven investigations conducted in the United States (43–49). This review included six studies from China (50–55). Further, three articles were from Poland, and two each from Ethiopia, Pakistan, Spain, Taiwan, and Turkey were included in this study. One article each was included from Australia, Bangladesh, Canada, Indonesia, Iran, the Republic of Korea, Mongolia, the Netherlands, Nigeria, Peru, Rwanda, the United Kingdom, and Sri Lanka (Figure 2).

Of the 41 studies that were included in the analysis, 10 (24.39%) were published in 2023, while the remaining 09 (21.95%) were reported in 2022. The year 2021 yielded the greatest number of eligible reports (12 out of 41; 29.26%) for this review article, whereas 8 out of 41 (19.51%) were published in 2020. Two eligible papers that meet the requirements for this scooping review eligibility were taken from 2019 publications (Table 1).

**Table 1:** General characteristics of included studies in scoping reviews (*n* = 41).

Characteristic	Number of studies	Percentage (%)
Publication year		
2023	10	24.39
2022	09	21.95
2021	12	29.26
2020	08	19.51
2019	02	4.87
Total	41	100
Study designs		
Cross-sectional study	32	78.04
Randomized Trial	5	12.19
Longitudinal study	2	4.87
Cohort Study	1	2.43
Non-randomized trial	1	2.43
Total	41	100
Type of disability reported in the study		
General disability	14	34.14
Physical disability	6	14.63
Intellectual	3	7.31
Migraine	3	7.31
Multiple sclerosis	3	7.31
Functional disability	2	4.87
Developmental disability	2	4.87
Chronic pain	2	4.87
Stroke	2	4.87
Cognitive impairment	1	2.43
Learning disability	1	2.43
Reading	1	2.43
Work disability	1	2.43
Total	41	100

Most of the studies included in this review were cross-sectional designs, with 32 (78.04%) spread across various countries around the world. All six studies from China, three from the United States and Poland, and two each from Ethiopia, Italy, Pakistan, Spain, Taiwan, and Turkey were cross-sectional in nature, focusing on outcome analysis at a specific time point during the study. Only 5 studies (12.19%) included randomized trials on people with disabilities, with two conducted in the United States and one each in the United Kingdom, Nigeria, and Iran. Furthermore, two longitudinal studies, one cohort, and another non-randomized trial were examined to determine depression in disabled people (Table 1).

Of the 41 studies, 14 reported an analysis of depression in individuals with disabilities but did not categorize them into types. Congenital disabilities such as spina bifida, cerebral palsy, and deformed limbs were present in a few of them. Visual, hearing, mobility, speaking, intelligence, and other mental defects have also been reported in general disabled people, either as individual difficulties or multiple disabilities in the same individual.

Six studies classified their samples as having physical disabilities, while three reports assessed samples with intellectual disabilities, migraine, and multiple sclerosis, respectively. Participants in two studies reported functional disability, developmental disability, chronic pain, and stroke. One study each reported on cognitive impairment, learning disability, reading difficulty, and work disabilities.

### Characteristics of the participants

The sample size ranged between 27 and 85,427. The farmer (56) was a cross-sectional study conducted in Mongolia to investigate the characteristics of multiple sclerosis and their correlation with depression levels, whereas the latter (57) was a report based on a national survey conducted in Indonesia to determine the mental health of physically disabled people. The average age of participants in the reviewed studies was 42 ± 16.19 (Mean ± SD), with a range of 10.94 to 74.4 years. All studies reported the participation of both male and female disabled people, except three studies (44, 45, 55), which did not report gender-based statistics, making it unclear whether they included participants of both genders. The average percentage of male and female participation in the remaining 38 studies was 46.01 and 53.58%. A study conducted in Mongolia by Jaalkhorol et al. (56) to determine the characteristics of multiple sclerosis and its correlation with depression level found the highest percentage of male participation (88.88%) and the lowest percentage of female participation (11.12%). In contrast, the highest percentage of female (92.2%) and the lowest percentage (7.8%) of male participation was observed in a study published by Irimia et al. (58) to analyze migraine and their association with anxiety and depression. Supplementary Table 1 contains a list of all 41 references, along with their study characteristics.

### Analysis of instruments used to measure depression

This review included studies that used 19 different types of instruments to measure depression in disabled people. Many studies used the patient health questionnaire (PHQ-9), which accounted for 29.26% (12 of 41), while six studies (6 of 41 = 14.63%) used Beck’s Depression Inventory (BDI), and three reports in this review used the hospital anxiety and depression scale (HADS). Center of Epidemiological Studies Depression Scale (CESD), Depression, anxiety, and Stress Scale (DASS)-21, Hamilton Depression Rating Scale (HAMD), and Zung Self-rating Depression Scale (SDS) were used by two studies each to measure depression in their respective study participants. A study (59) employed three different depression measuring tools [Beck Depression Inventory (BDI), Glasgow Depression Scale (GSD), and Signaallijst Depressie voor Zwakzinnigen (SDZ)] to analyze the outcomes of three screening instruments and compare the prevalence of depression in people with mild or borderline intellectual disability based on the measuring instrument. Children’s Depression Inventory (CDI) was used by a study (60) to evaluate the efficacy of Rational Emotive Behavior Therapy on Depression Among Children with Learning Disabilities. Another study conducted by Agnieszka et al. (61) relied on a doctor’s diagnosis rather than any specific standard instrument to assess the relationship between depression and the most common chronic diseases and disability in the older adult population of South-East Poland (the region of Podkarpackie). Depression scale-short form (GDS-15), Glasgow Depression Scale (GDS), Mini International Neuropsychiatric Interview questionnaire (MINI ICD-10), Negative Emotional Wellbeing Depression (NEWD), Neurological Depressive Disorders Inventory-Epilepsy (NDDI-E), Patient Health Questionnaire (PHQ-8), Patient-Reported Outcomes Measurement System (PROMIS) short-forms, Quality of Life in Neurological Disorders (Neuro-QoL), and Self-rating Depression Scale (SDS) were other depression measuring instruments used by at least one study included in this review (Table 2).

**Table 2:** Instrument/s used for measuring depression.

Number	Instrument	Number	Percentage
1.	Patient Health Questionnaire (PHQ-9)	12	29.26
2.	Beck’s Depression Inventory (BDI)	6	14.63
3.	Hospital Anxiety and Depression Scale (HADS)	3	7.31
4.	Center of Epidemiological Studies Depression Scale (CESD)	2	4.87
5.	Depression, anxiety, and stress scale (DASS)-21	2	4.87
6.	Hamilton Depression Rating Scale (HAMD)	2	4.87
7.	Zung Self-rating Depression Scale (SDS)	2	4.87
8.	Multiple tools [Beck Depression Inventory (BDI), Glasgow Depression Scale (GSD), and Signaallijst Depressie voor Zwakzinnigen (SDZ)]	1	2.43
9.	Children’s Depression Inventory (CDI)	1	2.43
10.	Depression scale-short form (GDS-15)	1	2.43
11.	Diagnosed by doctor	1	2.43
12.	Glasgow Depression Scale (GDS)	1	2.43
13.	Mini International Neuropsychiatric Interview questionnaire (MINI ICD-10)	1	2.43
14.	Negative Emotional Wellbeing Depression (NEWD)	1	2.43
15.	Neurological Depressive Disorders Inventory-Epilepsy (NDDI-E)	1	2.43
16.	Patient Health Questionnaire (PHQ-8)	1	2.43
17.	Patient-Reported Outcomes Measurement System (PROMIS) short-forms	1	2.43
18.	Quality of Life in Neurological Disorders (Neuro-QoL)	1	2.43
19.	Self-rating Depression Scale (SDS)	1	2.43
	Total	41	100

### Association of depression with disability

Although all 41 studies assessed depression in disabled participants, only 22 studies reported the percentage prevalence. Eight of them reported depression in general disability, which included hearing, visual, and speaking impairments. However, the prevalence of depression varied across these studies. A study conducted by Marti et al. in 2021 had all participants with depression. This study from the United States used a randomized trial to assess the relationship between changes in functional disability and suicidal ideation in older adults following depression psychotherapy. A cross-sectional study from Bangladesh reported by Roy et al. (62) found a 65.7% prevalence rate of depression among general disabled people. Another study, (48), measured depression in general disabled participants using the patient health questionnaire (PHQ-9) and discovered that 61% of them were depressed. A cross-sectional study of general disabled people in Ethiopia found a depression prevalence rate of 46.2% using a PHQ-9 measuring instrument. Barboza et al. (50), Bi et al. (63), and Pezzato et al. (64) reported depression prevalence rates of 43.3, 39.9, and 14.3%, respectively. Agnieszka et al. (61) evaluated depression in individuals with disabilities in general based on a physician’s diagnosis; they found a prevalence rate of only 8.06%. This was the lowest level of depression reported by researchers from Poland. As a result, in addition to disability, there may be several other factors that contribute to depression, either jointly or independently. Furthermore, the measuring tool and method of study design have a significant impact on the development of depression. Scheirs et al. (59) demonstrate the impact of measuring tools on determining the presence of depression in people with disabilities. They reported depression prevalence of 31, 44, and 22% using the Beck Depression Inventory, Glasgow Depression Scale, and Signaallijst Depressie voor Zwakzinnigen, respectively.

As shown by Table 3, all patients included in the multiple sclerosis study by Jaalkhorol et al. (56), work disability report by Sullivan et al. (65), developmental disability evaluation by McDermott et al. (66), learning disability determination by Ugwu et al. (60), functional disability report by Lutz et al. (45), general disability by Marti et al. (46), and physical disability determination by Zemestani et al. (67), had depression measured by different measuring instruments including PHQ-9, CDI, HAMD, and BDI in several parts of the world, namely, Mongolia, Canada, Spain, Nigeria, United States, and Iran.

**Table 3:** Prevalence of depression in different types of disability.

Number	Reference	Country	Type of disability	Study Design	Assessment tool for measuring depression	Prevalence of depression
1.	(56)	Mangolia	Multiple sclerosis	CROSS-SECTIONAL	Patient Health Questionnaire (PHQ-9)	100%
2.	(65)	Canada	Work disability	Non-randomized trial	Patient Health Questionnaire (PHQ-9)	100%
3.	(66)	Spain	Developmental disability	CROSS-SECTIONAL	Patient Health Questionnaire (PHQ-9)	100%
4.	(60)	Nigeria	Learning disability	Randomized Trial	Children’s Depression Inventory (CDI)	100%
5.	(45)	USA	Functional disability	cohort study	Hamilton Depression Rating Scale (HAMD)	100%
6.	(46)	USA	General disability	Randomized Trial	Hamilton Depression Rating Scale [HAMD]	100%
7.	(67)	Iran	Physical disability	Randomized Trial	Beck Depression Inventory (BDI)	100%
8.	(68)	Poland	Developmental disability	CROSS-SECTIONAL	Patient Health Questionnaire (PHQ-8)	70.80%
9.	(62)	Bangladesh	General	CROSS-SECTIONAL	DASS-21	65.70%
10.	(48)	USA	General disability	CROSS-SECTIONAL	Patient Health Questionnaire (PHQ-9)	61%
11.	(69)	Ethiopia	General disability	CROSS-SECTIONAL	Patient Health Questionnaire (PHQ-9)	46.20%
12.	(63)	Peru	General disability	CROSS-SECTIONAL	Patient Health Questionnaire (PHQ-9)	43.30%
13.	(70)	Ethiopia	Migraine	CROSS-SECTIONAL	Patient Health Questionnaire (PHQ-9)	41.40%
14.	(71)	Poland	Pain	CROSS-SECTIONAL	Patient Health Questionnaire (PHQ-9)	40.63%
15.	(50)	China	General	CROSS-SECTIONAL	Patient Health Questionnaire (PHQ-9)	39.90%
16.	(58)	Spain	Migraine LFEM	CROSS-SECTIONAL	Hospital Anxiety and Depression Scale (HADS)	39.30%
17.	(72)	Srilanka	Stroke	CROSS-SECTIONAL	Beck’s Depression Inventory (BDI)	35.80%
18.	(51)	China	Migraine	CROSS-SECTIONAL	Self-rating Depression Scale (SDS)	25.90%
19.	(64)	Italy	General disability	CROSS-SECTIONAL	Hospital Anxiety and Depression Scale (HADS)	14.30%
20.	(52)	China	Cognitive impairment	CROSS-SECTIONAL	Patient Health Questionnaire (PHQ-9)	8.60%
21.	(61)	Poland	General disability	CROSS-SECTIONAL	Diagnosed by doctor	8.06%
22.	(59)	Netherland	Intellectual, mild 73, borderline 29	CROSS-SECTIONAL	Beck Depression Inventory (BDI), Glasgow Depression Scale (GSD), and Signaallijst Depressie voor Zwakzinnigen (SDZ)	31% BDI, 44% GSD, 22% SDZ

### Interventions associated with depression

Out of the 41 studies that met the eligibility criteria, 14 included interventions. Five randomized trials were conducted, with one using a non-randomized approach to intervention. Furthermore, six of the studies involving interventions were cross-sectional in design, with one each using the longitudinal and cohort patterns (Supplementary Table 2).

Three studies reported interventions in people with general disabilities; two were cross-sectional, and one was randomized. Screen golf participation (73) and disabled people’s involvement in meaningful activities (47) were two interventions evaluated using a cross-sectional design, while a randomized trial comparing behavioral activation by licensed and lay counselors were conducted (46). Lutz et al. (45) used problem-solving therapy (PST) with functionally disabled people.

Cognitive behavior therapy (CBT) was used to treat people with developmental and learning disabilities (44, 60). Both studies found a statistically significant decrease in depressive symptoms after undergoing CBT. Another study (74) compared behavioral activation to guided self-help in people with intellectual disabilities. A study by Sullivan et al. (65) discovered that risk-targeted behavioral activation was an acceptable and effective intervention for work disabled.

Four of the interventional studies targeted people with physical disabilities. Tariq et al. (75) and Tariq et al. (76) conducted cross-sectional studies on perceived and cognitive social support, respectively. Zhao et al. (55) also investigated the effectiveness of perceived social support and resilience in the treatment of post-stroke depression. A randomized trial (67) used acceptance and commitment therapy (ACT). A longitudinal study (43) used a physical activity-based intervention. In a study on multiple sclerosis-disabled individuals, Carotenuto et al. (77) also employed interventions based on physical activities.

## Discussion

The purpose of this study was to determine depression among people with disabilities using published literature. For examination and additional elaboration, 41 research articles that met the eligibility criteria were chosen. These studies used varying measuring instruments to assess depression in people with various types of disabilities. Fourteen of the 41 studies examined depression in people with general disabilities, while only six of the selected reports focused on physically disabled people.

### Depression and disability

The published literature demonstrates that there is a correlation between specific types of disability and depression prevalence. Jaalkhorol et al. (56) found a 100% prevalence of depression among people with multiple sclerosis (MS), which could be attributed to MS-induced mental derangements or treatment-mediated depressive symptoms. This study found a direct correlation between corticosteroid treatment and depression levels in MS patients. This report was supported by previous research (78, 79), which found that corticosteroid administration causes depressive disorders, particularly among those receiving high-dose therapies. Developmental disability was also linked to a high prevalence of depression among adolescents (66). However, 57% of them had only minor depressive symptoms, while 33 and 10% had mild and moderate to severe symptoms, respectively. This study is consistent with previous findings (80), which estimated that 30–35% of all people with developmental disabilities have psychiatric disorders, with depression being the most prevalent. Cooper et al. (81) discovered that depression is the most common mental health issue among children with learning disabilities. This was documented by Ugwu et al. (60), in their study of Nigerian schoolchildren.

Another concerning condition that may lead to higher rates of depression (45) or higher levels of perceived burdensomeness or thwarted belongingness is functional disability, which is defined as difficulties carrying out daily activities (82). Similarly, the prevalence of clinically significant depression is well-linked to work-related musculoskeletal disorders (WRMD) (83, 84). Thus, literature provides strong support for the (65) report that shows a 100% prevalence rate of depression among people with disabilities at work.

The beginning and development of disability are linked to all degrees of depressive symptom severity, but severe depressive symptom severity is linked to a higher risk of severe and accelerated disability (24, 85–87). Disability tends to have a greater effect on depression than depression has on disability in these reciprocals and potentially spiraling relationships (88, 89). This is supported by Marti et al. (46), who discovered that people with general disability characteristics identified by the 12-item World Health Organization Disability Assessment Schedule (WHODAS 2.0) (90) exhibited varying depressive symptoms based on the degree of severity of disability.

### Depression and measuring instruments

It is also noted that there is variation in the prevalence rate of depression among similar types of disability studied in different parts of the world using disparate study designs and measurement tools. The wide variation in prevalence rates can be attributed to both differences and difficulties in methodology. This makes studies on the subject incomparable. To begin, prevalence can be expressed in three ways: as a single event, over some time, or a lifetime. When examining prevalence over an extended duration, rates naturally increase. Secondly, depression has been identified using a variety of measurement tools. Thirdly, sample variation could be the primary reason for the significant divergence in prevalence figures. There could be a distinction between people living with their families and those living in institutions, with the latter likely having the highest prevalence rates (38). Age and level of disability are two other factors that influence sample variety. There appears to be a link between increasing age and increased depressive symptoms (37). Research suggests that individuals with mild to moderate disabilities are more likely to experience depressive disorders (39, 40). Due to their incapacity to identify and communicate their thoughts and feelings, people with severe to profound disabilities may not exhibit depressive symptoms, which presents a measurement challenge (39).

The patient health questionnaire (PHQ-9) was found to be the most widely used depression measuring tool for a variety of disabilities across different parts of the world in most of the literature screened during this study (29.26%, 12 of 41). Participants in the PHQ-9 self-report questionnaire rate their feelings over the preceding 2 weeks using nine items. Typically, the questions were scored from 0 to 3, resulting in a range of 0 to 27 (0, not at all; 1, several days; 2, more than half the days; and 3, nearly every day). In addition, they classify it as moderate, ranging from 5 to 9; severe, ranging from 10 to 14 (91, 92). A cutoff of ≥10 is maintained in certain other studies for the diagnosis of major depression. According to Kroenke et al. (92), this cutoff has an 88% sensitivity and 88% specificity for diagnosing major depression. Beck’s Depression Inventory (BDI) is another frequently used tool for measuring depression. Designed to assess self-concept, anxiety, depression, anger, and disruptive behavior, this inventory kit was created by Beck et al. (93). Several studies (14.63%) included in this review used the Depression Inventory (BDI) from this kit. The BDI includes items that assess individual’s negative thoughts about themselves, their lives, and their future, including feelings of sadness and physiological signs of depression (94). On a four-point scale, participants are asked to rate how much they believe a sentence describes them (0 = never, 1 = sometimes, 2 = often, and 3 = always). In addition to the above, several other established depression diagnosis instruments were used; their validity and reliability are well documented; however, there is still variation in terms of depression severity outcomes. This suggests that while the majority of the instruments’ items were taken from the Diagnostic and Statistical Manual, some tests measure nearly the same aspects of depression while others might measure something slightly different. Given that different tests yield different percentages of individuals classified as depressed and that different tests do not identify identical cases, we propose that, as Scheirs et al. (59) showed in their study, employing a combination of tests is preferable to using any one test for diagnostic purposes.

### Interventions to mitigate depression

Moderation or timely intervention has been documented in 14 of the 41 studies to alleviate depression in people with disabilities. Most of the interventions were effective in reducing the severity of depressive symptoms and thus improving the quality of life for disabled people.

Depression in participants with physical disabilities was found to be lessened primarily by psychological counseling and mental health interventions. In the study published by Melville et al. (74), patients with intellectual disabilities received a 12-month behavioral activation and guided self-help intervention in separate groups. They concluded that the number of therapy sessions attended was directly related to positive outcomes in depression management, with no significant correlation between the two types of interventions.

Zemestani and Mozaffari (67) investigated the effects of eight weekly 90-min group sessions based on standard acceptance and commitment therapy (ACT) on physically disabled participants. Depression and other outcomes were measured at baseline, 8 weeks, and 16 weeks. After 8 weeks, the ACT group had significantly lower depressive symptoms than the control group. After 12 sessions of Problem-Solving therapy, people with functional disabilities showed positive results in terms of a significant decrease in depression and suicidal ideation (Lutz et al., 2022).

Bordeianu and Smith (44) discussed the use of cognitive behavioral therapy (CBT) to manage intellectual disability. They took a two-pronged approach, first providing an education module on depression recognition to the group home’s clinical staff and then offering weekly CBT to patients for 10 weeks. The findings revealed a significant increase in staff confidence and knowledge, as well as an improvement in depressive symptoms in the patients. Sullivan et al. (65) assessed risk-targeted behavioral activation for the treatment of depressed individuals who are work-disabled. The 10-week behavioral activation intervention was the main component of the treatment program, which also included strategies to address perceptions of injustice and catastrophic thinking, two psychosocial risk factors for delayed recovery. The study found that risk-targeted behavioral activation is an effective and acceptable intervention for managing depression in work-disabled people. In a study on learning-disabled students in schools, the level of depression significantly decreased in participants exposed to CBT at post-test and follow-up measures compared to those not exposed to it (60). Marti et al. (46) investigated whether behavioral activation in disabled individuals is associated with any treatment modality for depression. They provided behavioral activation by bachelor’s-level lay counselors (Tele-BA), problem-solving therapy by licensed clinicians (Tele-PST), and phone support calls (attention control). There were no significant differences between a lay counselor and a licensed clinician in decreasing depression among disabled individuals.

Tariq et al. (75) examine the role of perceived social support in physically disabled people and its impact on depressive symptoms. The perceived social support was assessed using a multidimensional scale of perceived social support (MSPSS). The study found that family and friend support was significantly associated with a decrease in depressive symptoms in participants with physical disabilities. Resilience and perceived social support were found to have a major influence on post-stroke depression in a study conducted by Zhao et al. in 2022. A 2019 study by Tariq et al. demonstrated the mediating role of cognitive social capital (reciprocity and interpersonal trust) in the association between depression and physical disability. This study discovered that a lack of interpersonal trust and reciprocity leads to a higher level of depression in people with disabilities. The research highlights the necessity of improving cognitive social capital interventions.

On the other hand, increasing physical activity is another intervention with positive implications for depression management. Battalio et al. (43) conducted a study on participants with one of four potential long-term physical disabilities (multiple sclerosis, muscular dystrophy, spinal cord injury, or postpoliomyelitis syndrome). The second stage of this longitudinal study was completed 3 years after the initial observations. They found that increased physical activity was associated with a decrease in depression severity over 3 years. Another study (77) on patients with multiple sclerosis found that physical exercise can reduce depression. In 2021, Bum et al. conducted a study that employed screen golf as a novel sport that can offer individuals with disabilities chances to participate in sports and have positive life experiences. They discovered that those who had played screen golf had significantly better mental health than those who had never played it.

Engaging in meaningful activities like walking, running, doing outdoor maintenance, reading, and socializing generally activates the body and stabilizes the mind. According to Oh et al. (47), a nationally reported survey found that more than half of older adults with disabilities participated in meaningful activities that effectively helped them manage their physical and mental disturbances including depression.

Overall, the results of this study highlight a significant issue that is frequently overlooked in the literature on mental health: adults with disabilities of any kind, regardless of age, are more likely to report having depressive symptoms. It has been observed that depressive symptoms, even if they do not progress to the point of major depression, nevertheless harm health (95). Moreover, cognitive decline can be predicted by depressive symptoms. Existing, limited research on access to mental health services for people with disabilities in general indicates that this population has more unmet needs (96). To mitigate the increased likelihood of depressive symptoms in individuals with disabilities, three primary approaches should be put into practice: (a) develop and implement policies to enhance the care of people with disabilities; (b) encourage routine symptom assessment and diagnosis of depression; (c) ensure that psychotherapy (97–99) facilities are accessible at all health centers to mitigate depression, including those in rural regions, that offer medical services to individuals with disabilities.

## Strengths and limitations

The scoping review was reported by PRISMA guidelines. Our team and the research librarian collaborated on the development of search terms. The necessary rigor and transparency are applied to the scoping review, which gathers data from studies with a variety of designs and methodologies. The data gathered from various study types for the scoping review is significant because it identifies the prevalence rate, depression measuring scale, methods of assessment, and intervention strategies for treating depression in individuals with disabilities. These findings will aid researchers in gaining a comprehensive understanding of the severity of depression and offer supplementary information for depression intervention in clinical practice. Additionally, there are a few limitations to this scoping review. We did not include important information from pertinent articles published in other languages because we limited our search to the literature published in English. This review did not conduct an RCT quality evaluation. Due to the small sample size, the population could not be fully represented. Therefore, to better understand depression rates and the intervention techniques used among people with disabilities in different parts of the world, we will broaden our search in future research to include publications published in other languages as well as monitor the emergence of new literature in this field. Because most of the studies included were cross-sectional in nature, we are unable to analyze depression status over time; thus, a longitudinal study is proposed to evaluate the extended or long-term effect of disability on depression.

### Recommendations

It is widely recognized that depression is one of the most prevalent psychological issues that have a detrimental effect on an individual, leading to a decrease in productivity, a reduction in everyday and professional functioning, financial difficulties, harm to relationships with others, and in extreme cases, death. The consequences of improper or delayed management action could be catastrophic and irreversible. Individuals with disabilities experience a greater degree of damage from depression compared to the general population. Consequently, effective strategies tailored to specific disabilities as well as successful interventions will be crucial in preventing depression and enhancing their quality of life.

## Conclusion

This scoping review identified 41 studies on the depression of people with disabilities published between 2019 and July 2023. Overall, all types of disabilities cause depression; however, the severity of depression varies depending on the level of difficulty disabled people face. Studies of people with similar disabilities revealed a highly diverse prevalence of depression rates. This variation could be attributed to different methods of diagnosing depression, administration methods, and/or study design. Based on the findings of the literature review, it is critical to use more than one method of measuring instruments to achieve more reliable results. Furthermore, all the intervention techniques described in the study were effective in lessening the intensity or symptoms of depression in people with disabilities. Regardless of the kind of disability, cognitive behavioral therapies and psychological counseling were widely used interventions that had notably positive effects in lowering the degree of depression. Some studies found that increasing physical activity, family support, and participation in meaningful activities reduced depression levels. More randomized controlled trials are needed, and they should focus on individuals with specific disabilities to provide disability-specific care that can enhance the quality of life that people with disabilities have about their health.

## Author contributions

SMA: Conceptualization, Formal analysis, Project administration, Supervision, Visualization, Writing – original draft, Writing – review & editing. SA: Conceptualization, Data curation, Writing – original draft. SAA: Conceptualization, Writing – original draft. AAA: Validation, Data curation, Writing – original draft, Software. AKA: Data curation, Writing – review & editing.
